# Deforestation within breeding ranges may still drive population trends of migratory forest birds in the East Asian Flyway

**DOI:** 10.1038/s41598-023-40626-3

**Published:** 2023-08-27

**Authors:** Jerome Chie-Jen Ko, An-Yu Chang, Ruey-Shing Lin, Pei-Fen Lee

**Affiliations:** 1 Taiwan Biodiversity Research Institute, Nantou, 552002 Taiwan; 2 Institute of Ecology and Evolutionary Biology, National Taiwan University, Taipei, 106216 Taiwan

**Keywords:** Ecology, Animal migration, Conservation biology, Forest ecology

## Abstract

The East Asian Flyway (EAF) is the most species diverse of global flyways, with deforestation in its migratory landbird’s non-breeding range suspected to be the main driver of population decline. Yet range-wide habitat loss impact assessments on EAF migratory landbirds are scarce, and seasonal variation in habitat preference of migratory species further increases the complexity for conservation strategies. In this study, we reviewed population trends of migratory forest breeding birds in the EAF along with their seasonal habitat preference from the literature and assessed the impact of forest cover change in species’ breeding and non-breeding ranges on population trends. We found that 41.3% of the bird species with trend data available are declining, and most have higher forest preference in the breeding season. Despite 93.4% of the species experienced deforestation throughout their annual cycle, forest cover change in the non-breeding range was not identified as the main driver of population trend. However, forest cover change in species’ regional breeding range interacts positively with the degree of breeding season forest preference in predicting population trends. We therefore stress that regional breeding habitat protection may still be important while following the call for cross-border collaboration to fill the information gap for flyway conservation.

## Introduction

Land-use change, including deforestation, is a major threat to global biodiversity, in addition to factors such as overexploitation, climate change, pollution, and invasive species^1^. Deforestation, in particular, has been linked to a severe decline in forest-dwelling species in biodiversity hotspots worldwide^2–4^. Migratory animals are particularly vulnerable to the effects of land-use change, due to the complexity of their biology and the conservation strategies required to protect them^5–7^. This complexity contributes to a faster decline in migratory species compared to non-migratory species^8^, with forest birds among the most severely impacted group^9^.

Deforestation in the non-breeding range of migratory forest breeding birds has long been suspected as a major contributor to population declines in the East Asian Flyway (EAF). The EAF is the most species-rich flyway globally, and also has the highest number of threatened species^10,11^. Forest loss and degradation are expected to be major threats to most migratory landbirds in the EAF that depend on forests as stopover and non-breeding habitats^11–13^. Deforestation in the EAF is among the most severe globally^14^, and has had a negative impact on the functional diversity of birds in the southern region^15^. For example, Borneo lost 34% (18.7 million hectares) of its old-growth forest between 1973 and 2015^16^. Additionally, migratory species may shift their habitat preferences between stages within their annual cycle^17–19^. Therefore, understanding the full-annual cycle status and threats, such as habitat loss, is critical to developing successful conservation strategies.

The understanding of threats affecting migratory landbird species at different stages of their annual cycle and range-wide population trends is an important knowledge gap that needs to be addressed globally^20^. Additionally, full life-cycle assessments often fail to take into account changes in seasonal habitat preferences between breeding and non-breeding ranges. Forest breeding species are particularly understudied, particularly in the EAF and tropical regions^21^. In the EAF, significant knowledge gaps include the impact of habitat conversion, loss, and degradation on assemblages of migratory landbirds in various habitats, including forests, particularly on their wintering grounds^11^. The lack of range-wide population monitoring efforts in the EAF further hinders the development of conservation strategies to address population decline, despite the high number of threatened species in the flyway^11^. While studies and efforts to address declines of migratory shorebirds and non-forest species (such as *Emberiza* buntings) in the flyway have increased in recent years^22,23^, an overall assessment of migratory forest birds in this highly threatened flyway is still lacking.

In this study, we reviewed population trends of migratory forest birds in the EAF and evaluated the impact of deforestation on both breeding and non-breeding ranges, taking into account changes in seasonal habitat preferences. Our objectives were to: (1) review existing regional population trends of migratory forest birds in the literature, (2) assess the trend of forest cover change in the species’ breeding and non-breeding ranges, (3) construct a seasonal, semi-quantitative forest habitat preference score for the study species, and (4) determine the relative importance of different drivers of population trends in relation to seasonal forest cover change and species’ forest habitat preference. By incorporating the species’ breeding and non-breeding seasonal forest preferences and forest cover changes, we aim to understand how deforestation in key stages of the species’ annual cycle affects the population status of migratory forest birds in East Asia.

## Results

### Trend of migratory forest breeding birds

**Figure 1:**
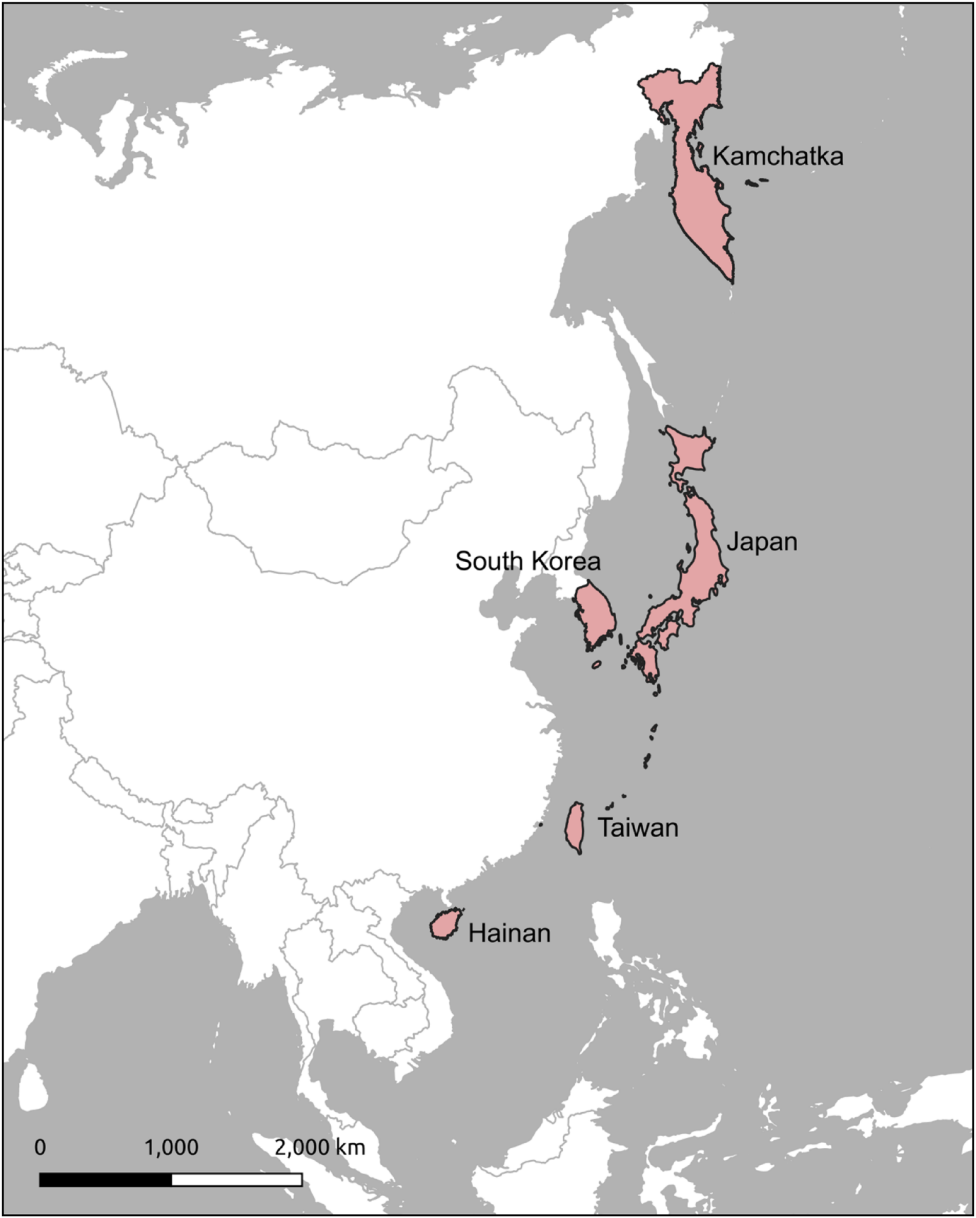
The extent of the East Asian Flyway (EAF) bounded by the 90th meridian on the west and the Pacific Ocean on the east, with the pink-colored areas marking our five study regions. The map was created using QGIS version 3.22.8 (https://qgis.org/en/site/).

**Figure 2:**
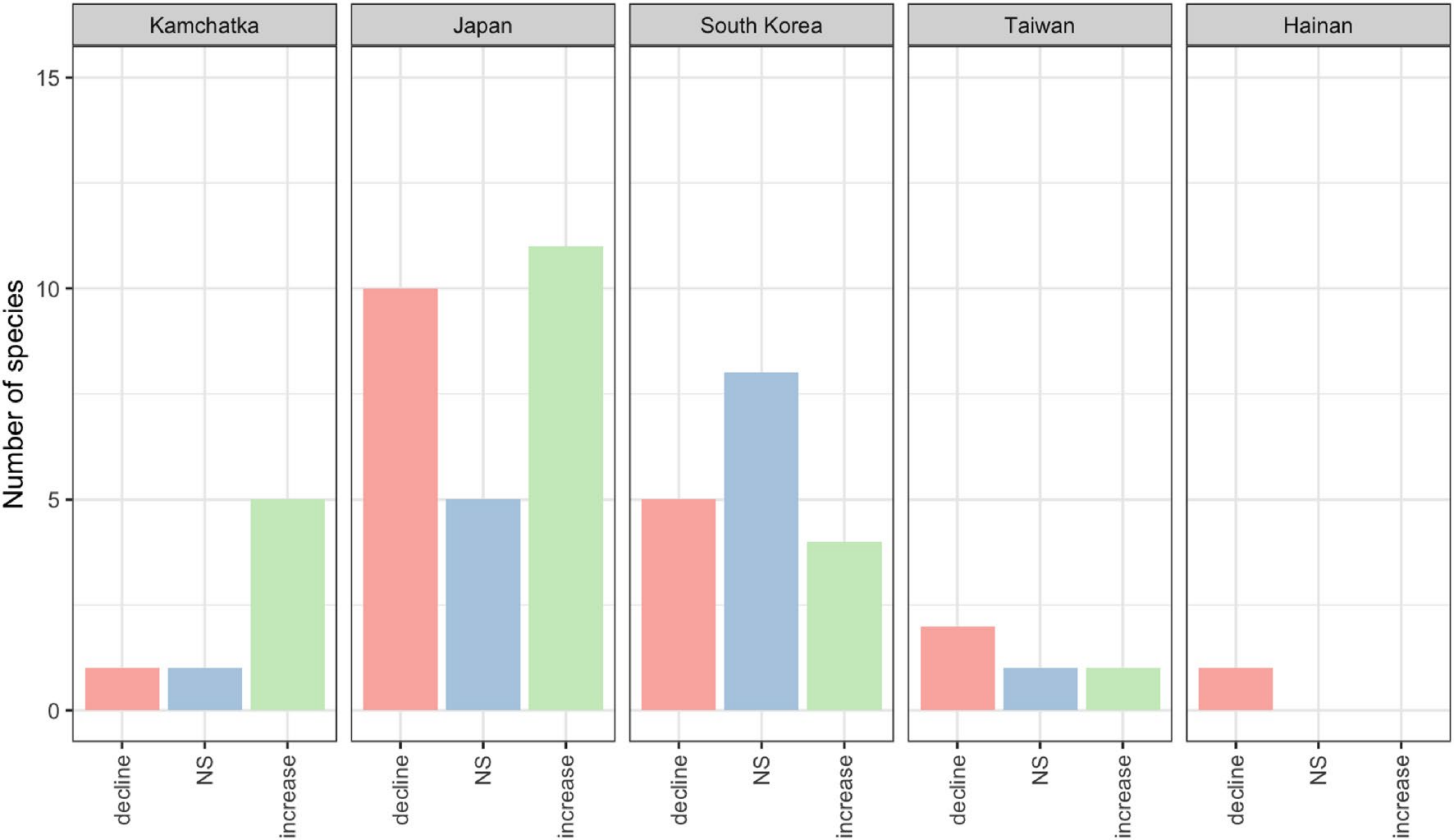
Number of species with declining, increasing or non-significant (NS) trends in the five regions from 46 species, 55 population trends compiled in this study.

We compiled a total of 46 migratory forest species with 55 regional breeding population trends within our five study regions from the literature (Fig. 1, Table 1). This represents approximately 20% of the 228 migratory forest land bird species in the East Asian Flyway (EAF)^11,24^. The population trends of these species were relatively evenly split, with similar numbers of species exhibiting an increasing trend (20 species, 43.4%) or a decreasing trend (19 species, 41.3%), and 11 species not having a significant trend reported (Table 2). Within each individual region, the number of increasing and decreasing species was similar, with the exception of Kamchatka, where the number of increasing species was four times higher than the number of decreasing species (Fig. 2).

**Table 1:** Summary of EAF migratory forest breeding birds' regional population trends compiled in this study.

Region	Literature	Year start/end	Year spans	Number of migratory landbirds species in this study (decline/NS/increase)	Data type	Trend information type
Kamchatka	Gerasimov and Lobkov^53^	1975/2018	44	7 (1/1/5)	Abundance	Text description in the literature
Japan	Ueta and Uemura^55^	1997/2021	25	26 (10/5/11)	Occupancy	Defined in this study
South Korea	Kim et al.^56^	1997/2015	23	17 (5/8/4)	Occupancy	Statistical analysis
Taiwan	Fan et al.^57^; Lin et al.^58^	2009/2020; 2001/2017	12; 17	4 (2/1/1)	Abundance	Statistical analysis
Hainan	Xu et al.^59^	1997/2013	17	1 (1/0/0)	Abundance	Statistical analysis

**Table 2:** Population trends of 46 species across the five regions in this study.

Scientific Name	Common Name	Kamchatka	Japan	South Korea	Taiwan	Hainan
*Acanthis flammea*	Redpoll	NS				
*Accipiter soloensis*	Chinese Sparrowhawk			▼ decline		
*Apus pacificus*	Pacific Swift		▼ decline			
*Butastur indicus*	Grey-faced Buzzard		▼ decline			
*Caprimulgus jotaka*	Grey Nightjar		△ increase			
*Coccothraustes coccothraustes*	Hawfinch		NS			
*Cuculus micropterus*	Indian Cuckoo			NS		▼ decline
*Cuculus saturatus*	Oriental Cuckoo		△ increase	NS	▼ decline	
*Cyanoptila cyanomelana*	Blue-and-white Flycatcher		△ increase	NS		
*Dendronanthus indicus*	Forest Wagtail			NS		
*Emberiza rustica*	Rustic Bunting	▼ decline				
*Emberiza sulphurata*	Yellow Bunting		△ increase			
*Eophona migratoria*	Chinese Grosbeak			NS		
*Falco subbuteo*	Eurasian Hobby		▼ decline	△ increase		
*Ficedula albicilla*	Red-throated Flycatcher	△ increase				
*Ficedula zanthopygia*	Yellow-rumped Flycatcher			▼ decline		
*Fringilla montifringila*	Brambling	△ increase				
*Geokichla sibirica*	Siberian Thrush		▼ decline			
*Halcyon coromanda*	Ruddy Kingfisher			▼ decline		
*Hierococcyx hyperythrus*	Northern Hawk Cuckoo		NS	▼ decline		
*Hierococcyx sparverioides*	Large Hawk Cuckoo				△ increase	
*Hirundapus caudacutus*	White-throated Needletail		▼ decline			
*Lanius tigrinus*	Tiger Shrike			△ increase		
*Larvivora akahige*	Japanese Robin		▼ decline			
*Larvivora cyane*	Siberian Blue Robin		▼ decline			
*Muscicapa dauurica*	Asian Brown Flycatcher		△ increase			
*Muscicapa ferruginea*	Ferruginous Flycatcher				NS	
*Muscicapa sibirica*	Dark-sided Flycatcher	△ increase				
*Oriolus chinensis*	Black-naped Oriole			NS		
*Passer cinnamomeus*	Russet Sparrow		NS			
*Pericrocotus divaricatus*	Ashy Minivet		△ increase			
*Pernis ptilorhynchus*	Oriental Honey-buzzard		▼ decline			
*Phylloscopus borealis*	Arctic Warbler	△ increase				
*Phylloscopus borealoides*	Sakhalin Leaf-warbler		△ increase			
*Phylloscopus coronatus*	Eastern Crowned Warbler		△ increase	△ increase		
*Phylloscopus tenellipes*	Pale-legged Leaf-warbler			NS		
*Phylloscopus xanthodryas*	Japanese Leaf-warbler		▼ decline			
*Pitta nympha*	Fairy Pitta		△ increase		▼ decline	
*Tarsiger cyanurus*	Orange-flanked Bush-robin		NS			
*Terpsiphone atrocaudata*	Japanese Paradise-flycatcher		△ increase			
*Turdus cardis*	Japanese Thrush		△ increase			
*Turdus chrysolaus*	Brown-headed Thrush		▼ decline			
*Turdus hortulorum*	Grey-backed Thrush			NS		
*Turdus obscurus*	Eyebrowed Thrush	△ increase				
*Turdus pallidus*	Pale Thrush			△ increase		
*Urosphena squameiceps*	Asian Stubtail		NS	▼ decline

### Seasonal forest cover change

**Figure 3:**
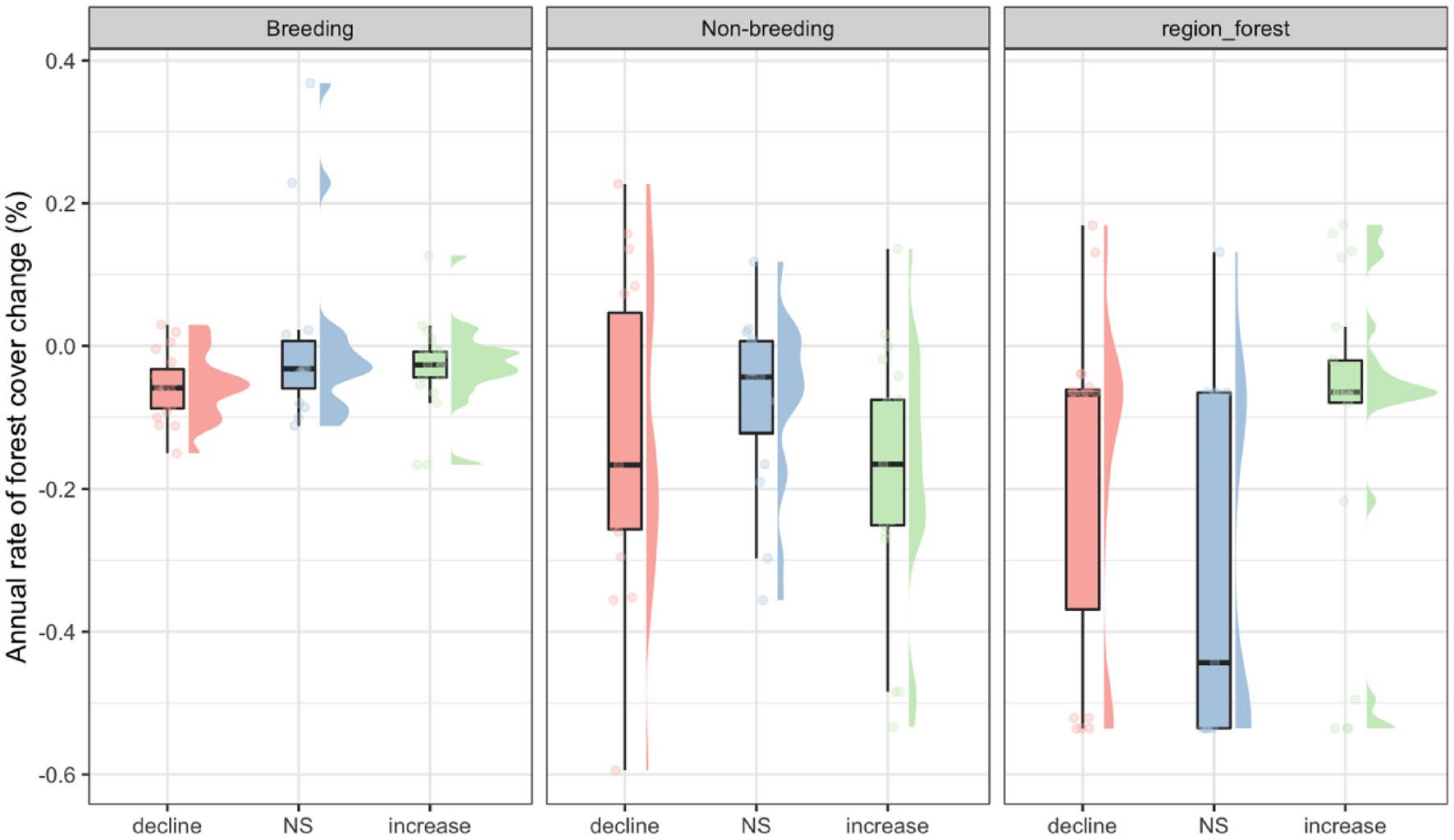
Annual rate of forest cover change of the three trend categories in breeding, non-breeding, and regional breeding ranges.

Our study revealed an overall deforestation across the 46 species' distribution range. The average annual rate (%) of forest cover change was −0.022 ± 0.087 and −0.138 ± 0.173 in the breeding and non-breeding range of the 46 species, respectively. Deforestation rate was significantly higher in the non-breeding range compared to the breeding range (paired Wilcoxon test, p = 0.0006, n = 46). The majority of species experienced forest cover loss in both their breeding and non-breeding ranges (28 species, 60.8%). Moreover, nearly all species (43 species, 93.4%) experienced forest cover loss in either their breeding or non-breeding range. However, the number of species that experienced forest cover loss in their breeding range was nearly the same as in their non-breeding range, with 35 and 36 species, respectively. For the 55 population trends, there was no significant difference in the annual rate of forest cover change between the increasing and declining trend categories in both the breeding and non-breeding seasons (Fig. 3). However, there was a higher variation in the annual rate of forest cover change among population trends in the non-breeding season compared to the breeding season, particularly with more severe declines (Fig. 3). After dividing the data into five regions, the patterns of forest cover change between trend categories were similar to the combined pattern (see Supplementary Fig. S1 online).

### Seasonal forest habitat preference change

**Figure 4:**
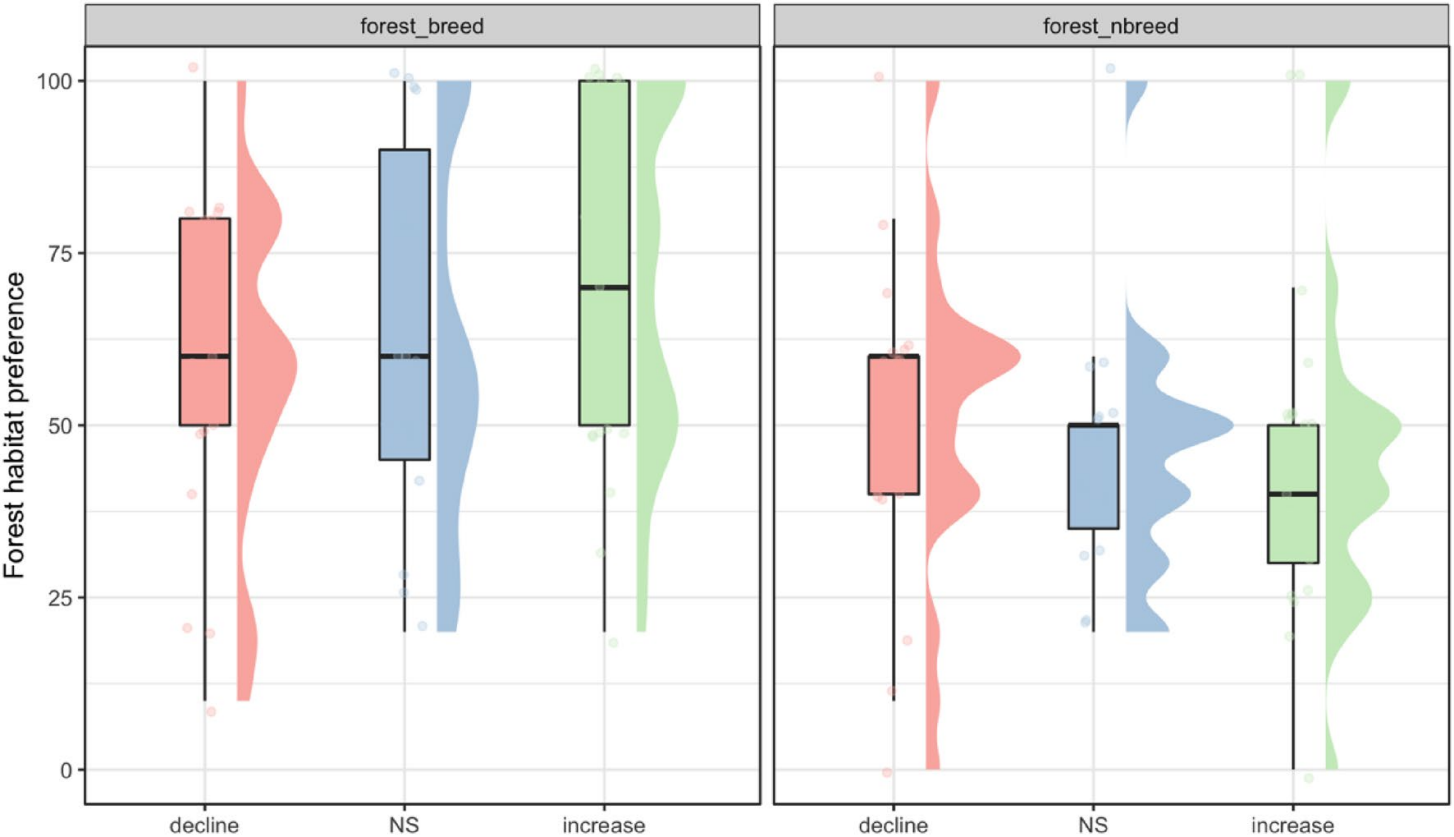
Scores of species’ forest habitat preference (0–100) of the three trend categories in breeding and non-breeding seasons.

We observed a significant shift in habitat preference from forest to non-forest habitats during the non-breeding season for the 46 species in our study. The mean forest habitat preference score for all species was 63.8 ± 26.8 in the breeding season, significantly higher than the mean score 45.8 ± 22.7 in the non-breeding season (paired Wilcoxon test. p = 0.00012, n = 46). Such pattern can be observed by that the majority of species (32 species, 69.6%) had a lower preference score for forest habitats in the non-breeding season. For the 55 population trends, visual examination of the data exhibits that species with higher forest habitat preference in the breeding season had increasing population trends, but this relationship was not observed in the non-breeding season (Fig. 4). This qualitatively assessed pattern was most pronounced in Kamchatka and Japan, but not as clear in other regions (see Supplementary Fig. S2 online). In South Korea, the data instead shows higher preference for forest habitats in the breeding season was instead associated with declining population trends (see Supplementary Fig. S2 online).

### Drivers of population trend

In the model selection process, we used a total of 36 migratory forest species with 40 regional population trends, after excluding those that were non-significant, to identify drivers of population trends. The global model, which included the forest cover change in each species' breeding range (B-FCC), non-breeding range (NB-FCC), and regional-breeding ranges (RB-FCC), and their interactions with the corresponding species' forest habitat preferences in the breeding (B-FPS) and non-breeding seasons (NB-FPS), accounted for 32.5% of the variability in the direction of regional population trends. All predictor variables were retained in the model due to their variance inflation factors (VIFs) being below 3. Additionally, model validation using the DHARMa package revealed no significant issues of overdispersion (Tables 3, 4).

**Table 3:** Predictor variables used in the generalized linear model.

ID	Predictor variable	Source
NB-FCC	Non-breeding range forest cover change	Forest cover change from ESA CCI LC^52^; Non-breeding range from BirdLife International^64^
B-FCC	Breeding range forest cover change	Forest cover change from ESA CCI LC^52^; Breeding range from BirdLife International^64^
RB-FCC	Regional breeding range forest cover change	Forest cover change from ESA CCI LC^52^; Regional species breeding range from BirdLife International intersected with target island (region)^64^
NB-FPS	Non-breeding season forest habitat preference	Forest habitat preference from Birds of the World^28^
B-FPS	Breeding season forest habitat preference	Forest habitat preference from Birds of the World^28^

**Table 4:** Candidate models for drivers of EAF migratory forest breeding bird's regional breeding population change.

ID	Variables	Driver hypotheses
mod1	NB-FCC	Non-breeding range forest cover change
mod2	B-FCC	Breeding range forest cover change
mod3	RB-FCC	Regional breeding range forest cover change
mod4	NB-FCC, B-FCC	Both non-breeding and breeding range forest cover change
mod5	NB-FCC, RB-FCC	Both non-breeding and regional breeding range forest cover change
mod6	NB-FCC, B-FCC, RB-FCC	Including non-breeding, breeding, and regional breeding range forest cover change
mod7	NB-FCC $\times$ NB-FPS	Mod1 with interaction between species' non-breeding forest habitat preference
mod8	B-FCC $\times$ B-FPS	Mod2 with interaction between species' breeding forest habitat preference
mod9	RB-FCC $\times$ B-FPS	Mod3 with interaction between species' breeding forest habitat preference
mod10	NB-FCC $\times$ NB-FPS, B-FCC $\times$ B-FPS	Mod4 with interaction between corresponding species' seasonal forest habitat preference
mod11	NB-FCC $\times$ NB-FPS, RB-FCC $\times$ B-FPS	Mod5 with interaction between corresponding species' seasonal forest habitat preference
Global model	NB-FCC $\times$ NB-FPS, B-FCC $\times$ B-FPS, RB-FCC $\times$ B-FPS	Mod6 with interaction between corresponding species' seasonal forest habitat preference

**Figure 5:**
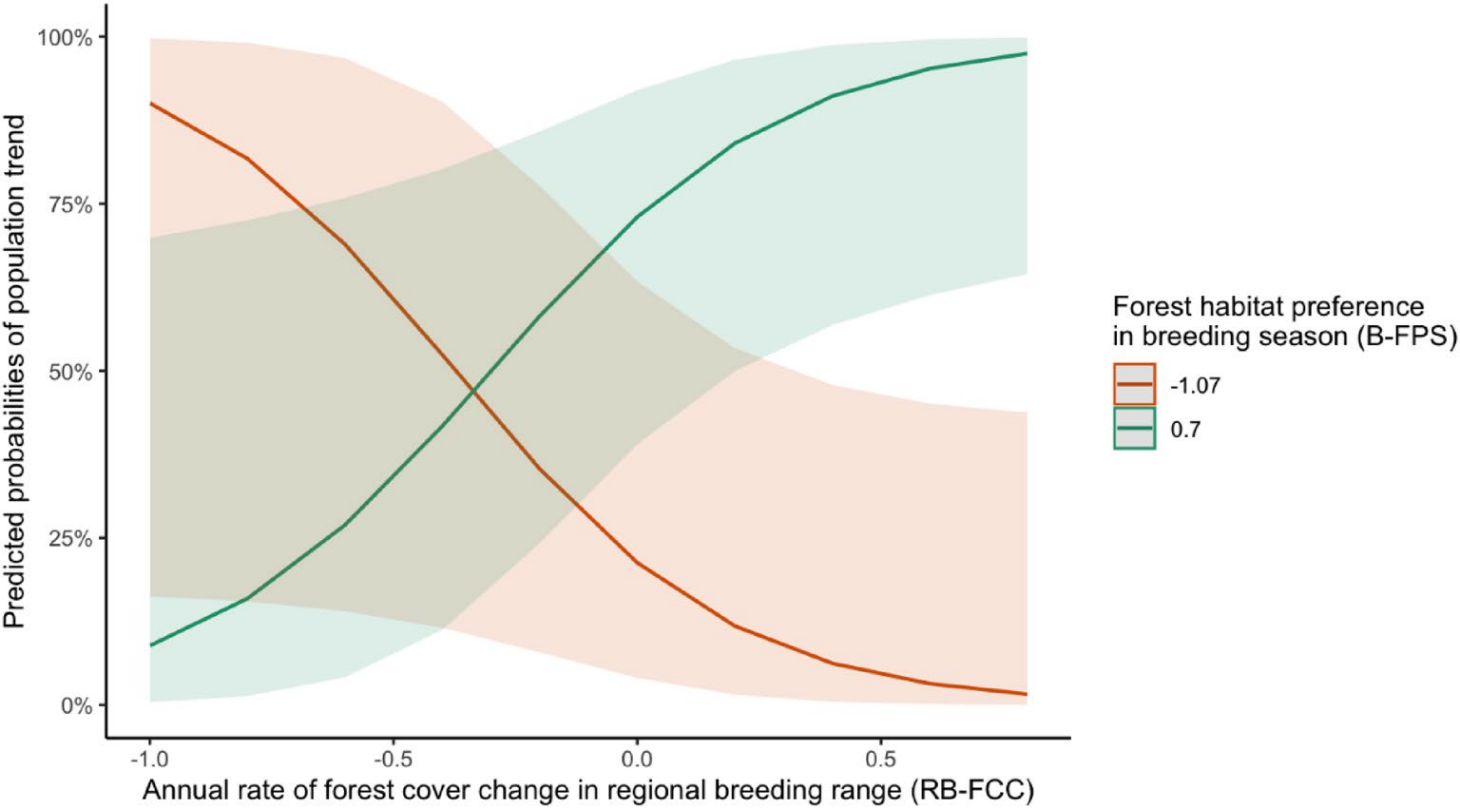
Marginal effect of the interaction between RB-FCC and B-FPS in predicting the probabilities of population trend outcome of EAF migratory forest breeding birds.

Among the candidate models, we found that only one model, incorporating the interaction between regional breeding range forest cover change (RB-FCC) and breeding season forest habitat preference (B-FPS), met the ΔAICc < 2 threshold and was therefore considered the best model for predicting species' regional population trend (Table 5). Model averaging also revealed that the interaction between RB-FCC and B-FPS was the only statistically significant variable in predicting population trend directions (Table 6, see Supplementary Fig. S3 online). This implies that an increasing population trend is more likely to occur when a species with higher forest habitat preference in the breeding season is also experiencing an increase in regional breeding forest cover change (Fig. 5). In contrast, models incorporating non-breeding range forest cover change (NB-FCC) with or without its interaction with non-breeding season forest habitat preference (NB-FPS) all had higher AIC scores than corresponding models with breeding range factors in the model selection process (Table 5) and were also non-significant in the model averaging results (Table 6).

**Table 5:** Model selection results for candidate models for drivers of EAF migratory forest breeding bird's regional breeding population change.

Variables	K	logLik	ΔAICc	*w*_*i*_
RB-FCC $\times$ B-FPS	4	−23.11	0.00	0.43
B-FCC	2	−26.61	2.19	0.14
RB-FCC	2	−27.25	3.47	0.08
NB-FCC, B-FCC	3	−26.13	3.56	0.07
NB-FCC	2	−27.33	3.62	0.07
B-FCC $\times$ B-FPS	4	−25.11	3.99	0.06
NB-FCC $\times$ NB-FPS, RB-FCC $\times$ B-FPS	7	−21.10	4.33	0.05
NB-FCC, RB-FCC	3	−26.79	4.89	0.04
NB-FCC, B-FCC, RB-FCC	4	−25.77	5.31	0.03
NB-FCC $\times$ NB-FPS	4	−26.14	6.06	0.02
NB-FCC $\times$ NB-FPS, B-FCC $\times$ B-FPS	7	−23.32	8.77	0.01
NB-FCC $\times$ NB-FPS, B-FCC $\times$ B-FPS, RB-FCC $\times$ B-FPS	9	−20.08	8.80	0.01

**Table 6:** Model averaging result for variables in all candidate models.

Variables	Estimate	SE	CI
None	0.10	0.35	(−0.60 to 0.80)
NB-FCC	−0.57	0.78	(−2.14 to 1.00)
B-FCC	1.09	0.77	(−0.47 to 2.66)
RB-FCC	0.62	0.75	(−0.90 to 2.13)
NB-FPS	−1.14	1.17	(−3.48 to 1.20)
B-FPS	1.36	0.92	(−0.50 to 3.23)
NB-FCC $\times$ NB-FPS	−1.31	3.31	(−7.98 to 5.36)
B-FCC $\times$ B-FPS	−1.32	2.01	(−5.41 to 2.76)
RB-FCC $\times$ B-FPS	3.97	1.85	(0.23 to 7.72)

## Discussion

In our analysis of EAF migratory forest birds, we found that 41.3% (19 species) of the species with available regional population trends have reported declining trends, while an equal proportion (43.5%, 20 species) have reported increasing trends. The majority of species (93.4%, 43 species) are experiencing deforestation in both their breeding and non-breeding ranges, with a higher rate of forest loss observed in the non-breeding range. However, most species (69.6%, 32 species) have a higher preference for forest habitats during the breeding season, indicating a lower association with forest habitats during the migration and winter. We found no evidence to support the speculation that forest cover change in the non-breeding range affects population trends. However, we found that the interaction between forest cover trends in regional breeding ranges and breeding season forest habitat preference is the best single model to predict trend direction. Specifically, increasing regional breeding population trends are more likely to occur when a species with a higher preference for forest habitats during the breeding season experiences an increase in forest cover in the corresponding region. We discuss these findings in more detail below.

The results of our literature search indicate that population trends of migratory landbirds in the EAF are indeed largely lacking, and integrating population status across studies is challenging. Out of the 228 migratory forest breeding bird species in the EAF, population trends that met our criteria for regional populations were only available for 46 species, which is c. 20% of the EAF migratory breeding bird species. Additionally, all studies, except the Taiwan Breeding Bird Survey, which is conducted annually, use a sampling scheme that repeats every 5–20 years or more, depending on the project. Furthermore, different data types and trend analysis methodologies across the literature further limit our ability to combine cross-regional study results into detailed population change information, such as percentage of population change per year. These limitations restrict our ability to test more complex hypotheses driving population change and do not provide sufficient information to distinguish between different levels of population change (e.g., moderate decline vs. strong decline). However, monitoring schemes that are sampled infrequently but regularly are reliable in determining trend signs^25^, therefore, we mainly rely on positive (increasing) or negative (declining) signs to compare different population change drivers. Our literature review also found that population trend reports were mainly on islands or peninsulas but not on mainland continents, which may have restricted our ability to describe a large proportion of migratory forest birds in the EAF. However, islands have been identified as having unique and important values for migratory birds globally^26^. Although the target species in this study are mainly common species that are not listed as threatened or near-threatened by the International Union for Conservation of Nature, they are long-distance migratory birds that may face multiple global change risks^27^. Considering the ongoing threat to migrating forest breeding birds posed by continuing deforestation in the EAF, we believe that the current data and methods used in this study can fill an important gap in understanding the relative importance of population change drivers throughout the annual cycle.

For the EAF migratory forest breeding birds in our study, we found that the majority of species have a higher preference for forest habitats during the breeding season compared to the non-breeding season. Migratory birds are known to have different associations with habitats between seasons, although the extent of species' niche tracking or switching on land cover types varies between migration seasons, regions, and species traits^17,27^. Despite emerging studies on this issue, species-specific habitat requirements data for migratory species in the EAF are still lacking^11^. Therefore, our study relied on seasonal habitat descriptions compiled from expert observations on the BOW online database^28^. By transforming the text descriptions from BOW into semi-quantitative information using a methodology commonly used in trait-related databases (e.g.,^29^), our study provides new seasonal habitat preference information for EAF migratory forest birds that is not currently available in regional and global bird trait databases (e.g.,^24,30,31^). Understanding seasonal habitat preferences is crucial for guiding conservation efforts for migratory species^32^. In our study, the effect of breeding range forest cover change on population change was strongest when interacting with the corresponding season's habitat preference. This result further demonstrates the potential usefulness of semi-quantitative habitat information in assessing the relationship between population dynamics and environmental change.

Despite higher rates of deforestation in the non-breeding ranges of EAF migratory species, we did not find evidence to support non-breeding range forest cover change as a significant predictor of increasing or declining population trends. This result contradicts previous speculations (cf.^11,12,33^), but also highlights the challenges in assessing the effects of non-breeding habitats on population changes of migratory species. One of the challenges is the coarse resolution and reliability of land use types classified by satellite imagery for identifying forest habitat change. For example, ground-truthed data occasionally reveals that habitats classified as "closed canopy forests" in land cover datasets may actually be human-modified habitats such as plantations or recently cleared land with little portions of native forest left^34^. Additionally, relying only on the "forest" habitat category for describing seasonal habitat change may limit our ability to detect the impact of habitat change on population trends. However, as most species diversify their habitat use in the non-breeding season in our study, this also implies the complexity of how habitat conversion affects migratory species in the wintering range, and supports the possibility that deforestation may not be the only main driver. Notably, since our understanding of habitat preference and the function of each habitat in the non-breeding range is less developed for migratory birds, especially when compared to our knowledge of the strong relationship with breeding requirements during the breeding season, future research in this area should be encouraged.

The level of migratory connectivity, which describes how a species' population redistributes between its breeding and non-breeding ranges, may also affect the ability to find consistent effects of non-breeding habitat loss on species population trends. Recent studies have shown that if migratory connectivity is moderate or low between non-breeding and breeding sites^35,36^, the effect of habitat loss on certain non-breeding sites may be buffered by the mixing of individuals from other non-breeding sites^37^. Moderate migratory connectivity does seem to be common^38^, and low migratory connectivity is especially thought to be more common in long-distance migratory birds^39^. In the EAF, very few studies of migratory connectivity in migratory forest breeding birds have been conducted. An exception is a recent study on the Siberian Rubythroat (*Luscinia calliope*), which is a shrubland breeding migratory species also seeming to show low migratory connectivity^40^. However, as such information is still largely lacking, this limitation further supports the need for more studies in the wintering range of migratory landbirds in the EAF.

The effect of regional breeding forest cover change on migratory species in our study area may also be surprising, as most of our study regions are expected to be doing well in protecting or restoring forest, especially when compared to the era before the mid-twentieth century (e.g.,^41,42^). However, despite a relatively low rate, forest loss is still occurring in our study regions. This pattern is not only observed in the ESA CCI LC land use data we used, but also supported by data from the Global Forest Watch^14^. For our five study regions, Kamchatka has a 1.4% (251 kha) loss of forest between 2000 and 2019, Japan a 2.7% (714 kha) loss, South Korea a 5.4% (288 kha) loss, Taiwan a 1.8% (41.4 kha) loss, and Hainan a 15% (264 kha) loss, respectively^43^. Our results therefore show that despite forest conservation efforts after the mid-twentieth century, more studies are needed to be conducted in these regions to provide better breeding habitat protection for halting migratory species decline. Indeed, cases have shown that protecting breeding ranges is still important and effective in preventing further population decline^44–46^. This may be achieved by protecting specific breeding regions that will have the effect of sustaining populations in all breeding regions by preventing shifts in migratory connectivity^47^. Prioritizing sites to support migratory species based on both connectivity and abundance are effective strategies to be applied^48^. Overall, these conservation strategies will require ongoing financial support for population monitoring in the breeding region to suggest the most effective target locations. In recent years, it's encouraging to see an increasing number of studies published on the status of migratory land bird breeding populations in the EAF (Table 1). However, many regions still lack this information, considerably so in the continental East Asia^49^. Future monitoring efforts should ideally not only provide abundance metrics but also demographic metrics, with an emphasis on reporting site-level population statuses^46^.

As the trend of deforestation continues to decline in the EAF, accompanied by a 41.3% of known migratory forest breeding species declining, we follow the call of migratory shorebirds and buntings to stress the need for cross-border collaboration to research and conserve the diversity of the flyway^11^. Although we did not consider the effect of climate change on migratory forest breeding birds in the EAF, migratory species may be more susceptible to habitat loss especially for long-distance migrants because of low migratory connectivity^39^. Hunting is also an increasingly recognized threat affecting survival rates in the non-breeding region of migratory birds in the EAF^50,51^. While future research will be required to address both climate change and hunting impacts on the population status of EAF migratory land birds, suggestion of current mitigation efforts are already necessary to halt the decline of these species. Our results highlight the importance of regional habitat protection efforts in northern countries, as their conservation efforts may still play a crucial role in mitigating population decline.

## Methods

### Population trend data

We compiled population trends of migratory landbird species from published literature that assess regional bird population status change in the EAF. We conducted literature search through the Web of Science (www.webofscience.com) by combining the following search terms: “east asia bird”, “population trend”, “decline”, “change”. Studies published between 1990 and 2021 were all screened. From the studies we found and their citing literature, we further located additional related studies. We also used the same search term in Google Scholar and compiled all the literature available. Both abundance-based (e.g., density) and occupancy-based (e.g. breeding bird atlas) studies were included in our scope. Only studies with a monitoring time frame more than 10 years after 1992 were included. This requirement is set to fit our land cover data which started in 1992^52^. If multiple population trends for one species were given within one region (e.g. many studies in Japan), for every species we only retain one study in one region, by filtering out the one with the largest geographical and longest temporal coverage. Our literature search resulted into the following eight studies, with one study in Kamchatka, Russia^53^; two studies in Japan^54,55^; two studies in South Korea^23,56^; two studies in Taiwan^57,58^; and one study in Hainan Island^59^. From these studies, we filtered out 386 EAF migratory landbirds species following the definition in^11^. From the previous 386 species we further filtered out 228 forest bird species (including forest and woodland species) according to the definition by^24^. Finally, to focus on migratory breeding population trends of each species, only population trends from regions where our target breeds without resident populations (i.e. summer visitors only) were used in this study. Definition of species’ regional migratory status is based on each region’s available trait database (^30^ for Japan,^31^ for Taiwan) or checklist reviews (^60^ for Kamchatka,^61^ for South Korea,^62^ for Hainan Island).

From each study, we assigned the population change result of each species into three trend categories: “decline”, “increase”, and “non-significant”. If the reference provides species-specific population trends based on either statistical trend analysis or local expert opinion, we directly adopt the trend type given by the study. In cases where no species-specific result was provided, we categorized the trends by the following protocol. First, for occupancy studies (e.g.,^55^), we use the following formula to calculate the ratio of occupancy change:

$$Ratio\, of\, occupancy\, change \left(\%\right)=\frac{{{N}_{end }-N}_{start}}{{N}_{start}}$$

where N is the number of occurring grids. Species with ratio of occupancy change over 10% were assigned into the “increase” trend category, those with ratio lower than −10% were assigned into the “decrease” category, while the rest are assigned into the “non-significant” category. We adopted the 10% criteria following the IUCN Red List Categories and Criteria for C1 criteria^63^. Secondly, for abundance studies, if the average density and occupancy across all survey sites at the end of the given time interval are either 10% higher or lower those at the initial time, the species is assigned an “increase” or “decrease” trend, respectively. Furthermore, if only one of these metrics (either average density or occupancy) changes beyond the 10% threshold, while the other remains within it, the species is still assigned and “increase” or “decrease” trend, based on the metric that changed. In cases where neither metric surpasses the 10% change threshold, or when both metrics surpasses the threshold but in opposite directions, the species is classified as having a “non-significant” trend. Only species with an average of over 10 samples presence (either occupancy grids or survey plots) between the time interval of the study were assigned with a trend category.

### Seasonal forest cover change

To obtain the change in forest cover within the distribution ranges of each species, we used global land cover data from the European Space Agency Climate Change Initiative Land Cover (ESA CCI LC). The ESA CCI LC provides global land cover data, gathered between 1992 and 2019, with a resolution of 300 m^52^. We defined the area of forest cover by combining tree cover and mosaic tree-related land cover types present in the EAF in the ESA CCI LC (see Supplementary Table S1). For the species distribution ranges, we defined three types: breeding range, non-breeding range, and regional breeding range. We used the definition of range status from BirdLife International for each species' breeding and non-breeding ranges^64^. The regional breeding range is confined to the area where the corresponding regional breeding population trend was obtained. We calculated the trend of percentage forest cover change in each species' ranges using simple OLS regression. We used percentage forest cover change instead of the exact area change to focus on the proportion of change in each species' corresponding range. The temporal range of each population trend was calculated using data from the year before the starting year, to data from the year before the ending year. For example, if the population trend is from 2009 to 2020, we obtained corresponding forest cover change from land cover data within 2008 to 2019. The resulting forest cover change trend will also be used in the following driver analysis.

### Seasonal forest habitat preference

To obtain semi-quantitative information on habitat preference across breeding and non-breeding seasons of EAF migratory land birds, we applied a defined protocol to standardize qualitative habitat descriptions from the literature. This protocol was largely borrowed from^29^, where the authors created a database of species-level foraging attributes of birds and mammals by extracting information from expert descriptions in the literature. We used a consistent method to translate qualitative habitat descriptions into quantitative scores. For each habitat category, we assigned a score of 0 to 100, representing an estimated percent relevance (with a minimum score of 5 for each category). The total value of all habitat categories for a given season was 100.

We used the Birds of the World database of Cornell Lab of Ornithology (BOW) as a reference for habitat categorization for both breeding and non-breeding seasons of all species in our study. Data from the BOW were accessed between November 2021 and July 2022. We recognized a total of 16 habitat types (see Supplementary Table S2 online) based on the first level of the IUCN habitat classification scheme Version 3.1^65^. The only exception was in the Artificial—Terrestrial type, where we also recognized its second-level habitat types, such as arable land, plantations, and urban areas. We believe that distinguishing between these second-level habitat types will avoid confusion as they represent very different habitat textures, particularly for forest-breeding birds. However, the second-level habitat type in Artificial—Terrestrial "14.6 Subtropical/Tropical Heavily Degraded Former Forest" was assigned to the "Forest" type, as it is not distinguishable based on the habitat description in the BOW text and may not be distinguishable to forest migratory birds. Examples of the logic used to translate descriptions into standardized numerical values include: "frequents forest" or “favors grassland”: score 50 or higher; "also occurs in shrub": score 10 or 20; "occasional in urban areas" or “possibly in wet meadows”: score 10 at most. If many habitat types are listed as being significant, we decreased the score of each. In cases where multiple terminologies could be categorized as one habitat type, they were first combined and then given the score according to the highest habitat preference description. In cases where comparative words were not used in the description for a certain habitat, we gave higher scores to the first listed habitat type, then decreasing proportions for those following. Breeding and non-breeding season’s habitat type scores were given separately, but if descriptions were not differentiated in the reference, both seasons were given identical habitat categorization scores. Additionally, for the non-breeding season’s habitat type, we disregarded descriptions on habitat recorded during migration if they were indicated to differ from the wintering season ranges. This is because habitat use during migration may be more opportunistic and not necessarily strongly related to survival, and that our focus is primarily understanding the effects from breeding and wintering ranges. To ensure uniformity and consistency of judgment, all translations of description to habitat scores were done by one of the first author of this study and reviewed by the third author. Results of habitat categorization are available in the Supplementary material (see Supplementary Table S3 online).

In our study, for both breeding and non-breeding seasons, we only use the forest habitat preference score for the “forest” category for each species in the following analysis.

### Drivers of population trend

To assess the relative importance of various drivers of population trends in relation to seasonal forest cover change and species' forest habitat preference, we employed generalized linear models with binomial distribution for the trends of the regional population of the study species. The model response was either 1 (increase) or 0 (decrease). We excluded regional population trends with non-significant trends as they provide ambiguous information and may indicate either stable or specific trends that are undecidable due to data limitations. The predictor variables in these models include variables that represent forest cover changes in each species' breeding (B-FCC), non-breeding (NB-FCC), and regional-breeding ranges (RB-FCC) and variables that represent the species' forest habitat preferences in breeding (B-FPS) and non-breeding seasons (NB-FPS) (Table 3).

We ranked all candidate models by the Information-Theoretic approach^66^, using corrected Akaike’s Information Criterion (AICc) that accounted for small sample sizes. The candidate models represented alternative drivers of population trends, with the global model containing interactions between forest cover change of all three types of species' ranges and their corresponding season's forest habitat preference. Specifically, the variables included in the global model were interactions between B-FCC and B-FPS, NB-FCC and NB-FPS, and finally RB-FCC and B-FPS. All candidate models (n = 14) represented drivers that considered different combinations of seasonal habitat change, with or without interactions between their corresponding season's forest habitat preference (Table 4). For model averaging we used the zero method since we aimed to find the driver with the most substantial effect on the population trend^67^. We calculated R^2^ of the global model using the Tjur’s R^2^, which represents the difference in the mean predicted probabilities of a binary dependent variable^68^. For assessing multicollinearity among predictor variables, we used package ‘car’^69^, and eliminated predictor variables if their variance inflation factors (VIFs) were above 3^70^. Since we have interaction terms in our model, to avoid impaired interpretation of the effects of predictor variables, we standardized all the input variables to a mean of 0 and a SD of 0.5, following the suggestion by^67^. We used the 'DHARMa' package for model validation and to evaluate residual diagnostics^71^. All statistical analyses were performed using R software^72^.

### Supplementary Information

Supplementary Information.

## Data Availability

Data supporting the results of the study can be accessed upon reasonable request from the corresponding author.
