# Supplementary material for: Deforestation within breeding ranges may still drive population trends of migratory forest birds in the East Asian Flyway
Source: Sci Rep. 2023 Aug 27;13:14007. doi: 10.1038/s41598-023-40626-3 (PMC10460791; doi:10.1038/s41598-023-40626-3)
Supplement: Supplementary file 1 — Supplementary Information. [file 41598_2023_40626_MOESM1_ESM.docx]

**Supplementary material**

**Table S1.** Land cover types from ESA CCI LC that are classified as forest habitat in this study.

| ESA CCI LC Value | Label |
| --- | --- |
| 50 | Tree cover, broadleaved, evergreen, closed to open (>15%) |
| 60 | Tree cover, broadleaved, deciduous, closed to open (>15%) |
| 61 | Tree cover, broadleaved, deciduous, closed (>40%) |
| 62 | Tree cover, broadleaved, deciduous, open (15‐40%) |
| 70 | Tree cover, needleleaved, evergreen, closed to open (>15%) |
| 71 | Tree cover, needleleaved, evergreen, closed (>40%) |
| 72 | Tree cover, needleleaved, evergreen, open (15‐40%) |
| 80 | Tree cover, needleleaved, deciduous, closed to open (>15%) |
| 81 | Tree cover, needleleaved, deciduous, closed (>40%) |
| 82 | Tree cover, needleleaved, deciduous, open (15‐40%) |
| 90 | Tree cover, mixed leaf type (broadleaved and needleleaved) |
| 100 | Mosaic tree and shrub (>50%) / herbaceous cover (<50%) |
| 160 | Tree cover, flooded, fresh or brakish water |
| 170 | Tree cover, flooded, saline water |

**Table S2.** Categories of the 16 habitat types with definitions

| **No.** | **Habitat type** | **Definition (IUCN 2021 [62])** |
| --- | --- | --- |
| 1 | Forest | Forest consists of a continuous stand of trees and includes both forested areas (generally with a closed canopy) and wooded areas (canopy more open, but see 2. Savanna). |
| 2 | Savanna | Savannas are transitional between grasslands and forests. They are ecosystems dominated by a grass ground cover with an overstorey of widely spaced trees and shrubs. May be referred to as savanna woodlands, savanna parklands, savanna grasslands, low tree/shrub savannas, thicket/scrub savannas. |
| 3 | Shrubland | Also referred to as scrub, bushland and thicket. |
| 4 | Grassland | Grasslands occur in regions with warm growing seasons and moderate water shortages. Native grasslands are comprised of grasses and broadleaved herbaceous plants, and are either without woody plants, or the latter are very sparsely distributed (see also 2. Savanna). |
| 5 | Wetlands(inland) | Inland wetlands correspond to the wetland types recognized by Ramsar. |
| 6 | Rocky Areas | Includes inland cliffs, mountain peaks, talus, feldmark |
| 7 | Desert | Desert consists of arid landscapes with a sparse plant cover, except in depressions where water accumulates. The sandy, stony or rocky substrate contributes more to the appearance of the landscape than does the vegetation. |
| 8 | Marine - Neritic | Submergent (below extreme low tide), nearshore, on or over the  continental shelf or oceanic island shelf. |
| 9 | Marine - Intertidal | Area of the shore between the extremes of high and low tides. |
| 10 | Marine - Coastal/Supratidal | Coastal habitats above the high tide mark. These largely match to the coastal habitats used by Ramsar. |
| 11 | Artificial - Arable Land | Includes cereal fields, rice paddies, perennial crops, orchards and  groves. |
| 12 | Artificial - Pastureland | Includes fertilized or re-seeded permanent grasslands, sometimes  treated with selective herbicides, with very impoverished flora and  fauna. Also includes secondary grasslands and wooded farmland. |
| 13 | Artificial - Plantations | A plantation is an intentional planting of a crop, on a larger scale,  usually for uses other than cereal production or pasture. The term is currently most often used for plantings of trees and shrubs. The term tends also to be used for plantings maintained on economic bases other than that of subsistence farming. Plantations are typically (but not exclusively) found in tropical or semitropical countries and usually require resident labourers. |
| 14 | Artificial - Rural Gardens | Rural gardens are located in a rural setting, serving families whose  main income comes from wage labour (rural or urban). |
| 15 | Artificial - Urban Areas | Occurs throughout the world. Usually metropolitan and commercial areas dominated by asphalt, concrete and roof. Includes buildings, lawns and parks. |
| 16 | Artificial Aquatic | These are human-made wetland habitats. |

**Table S3.** Results of habitat categorization of the 46 migratory forest breeding bird species in this study. The scores of breeding season and non-breeding season habitat preference were divided by “/” in each category. From the original 16 habitat types, Desert, Marine Neritic, Marine Intertidal, Artificial Aquatic were not shown since none of the 46 species preferred these habitat types in both seasons.

| Latin name | Forest | Savanna | Shrubland | Grassland | Wetlands (inland) | Rocky Areas | Marine Coastal/ Supratidal | Artificial - Arable Land | Artificial - Pastureland | Artificial - Plantations | Artificial - Rural Gardens | Artificial - Urban Areas |
| --- | --- | --- | --- | --- | --- | --- | --- | --- | --- | --- | --- | --- |
| *Acanthis flammea* | 20 / 20 | 0 / 0 | 40 / 40 | 40 / 40 | 0 / 0 | 0 / 0 | 0 / 0 | 0 / 0 | 0 / 0 | 0 / 0 | 0 / 0 | 0 / 0 |
| *Accipiter soloensis* | 60 / 60 | 0 / 0 | 0 / 0 | 0 / 0 | 10 / 10 | 0 / 0 | 0 / 0 | 10 / 10 | 0 / 0 | 20 / 20 | 0 / 0 | 0 / 0 |
| *Apus pacificus* | 10 / 10 | 10 / 10 | 10 / 10 | 10 / 10 | 10 / 10 | 10 / 10 | 10 / 10 | 10 / 10 | 0 / 0 | 0 / 0 | 10 / 10 | 10 / 10 |
| *Butastur indicus* | 20 / 0 | 0 / 0 | 20 / 0 | 0 / 0 | 20 / 0 | 0 / 0 | 0 / 0 | 20 / 50 | 20 / 50 | 0 / 0 | 0 / 0 | 0 / 0 |
| *Caprimulgus jotaka* | 50 / 50 | 0 / 0 | 50 / 20 | 0 / 0 | 0 / 0 | 0 / 0 | 0 / 0 | 0 / 0 | 0 / 0 | 0 / 20 | 0 / 0 | 0 / 10 |
| *Coccothraustes coccothraustes* | 40 / 20 | 0 / 0 | 15 / 20 | 0 / 0 | 0 / 0 | 0 / 0 | 0 / 0 | 15 / 20 | 0 / 0 | 0 / 0 | 15 / 20 | 15 / 20 |
| *Cuculus micropterus* | 60 / 60 | 0 / 0 | 0 / 0 | 0 / 0 | 0 / 0 | 0 / 0 | 0 / 0 | 40 / 40 | 0 / 0 | 0 / 0 | 0 / 0 | 0 / 0 |
| *Cuculus saturatus* | 50 / 40 | 0 / 20 | 30 / 0 | 0 / 0 | 0 / 10 | 0 / 0 | 0 / 0 | 20 / 10 | 0 / 0 | 0 / 10 | 0 / 10 | 0 / 0 |
| *Cyanoptila cyanomelana* | 60 / 50 | 0 / 0 | 20 / 0 | 0 / 0 | 0 / 0 | 0 / 0 | 0 / 0 | 0 / 0 | 0 / 0 | 20 / 50 | 0 / 0 | 0 / 0 |
| *Dendronanthus indicus* | 100 / 30 | 0 / 0 | 0 / 0 | 0 / 0 | 0 / 10 | 0 / 0 | 0 / 0 | 0 / 20 | 0 / 0 | 0 / 20 | 0 / 20 | 0 / 0 |
| *Emberiza rustica* | 60 / 40 | 0 / 0 | 20 / 20 | 0 / 0 | 20 / 20 | 0 / 0 | 0 / 0 | 0 / 20 | 0 / 0 | 0 / 0 | 0 / 0 | 0 / 0 |
| *Emberiza sulphurata* | 80 / 0 | 0 / 0 | 0 / 30 | 0 / 40 | 0 / 0 | 0 / 0 | 0 / 0 | 0 / 30 | 0 / 0 | 0 / 0 | 20 / 0 | 0 / 0 |
| *Eophona migratoria* | 50 / 50 | 0 / 0 | 0 / 0 | 0 / 0 | 20 / 20 | 0 / 0 | 0 / 0 | 10 / 10 | 0 / 0 | 0 / 0 | 10 / 10 | 10 / 10 |
| *Falco subbuteo* | 20 / 60 | 0 / 30 | 20 / 0 | 0 / 0 | 20 / 0 | 0 / 0 | 0 / 0 | 10 / 0 | 10 / 0 | 0 / 0 | 10 / 10 | 10 / 0 |
| *Ficedula albicilla* | 100 / 40 | 0 / 0 | 0 / 0 | 0 / 0 | 0 / 0 | 0 / 0 | 0 / 0 | 0 / 0 | 0 / 0 | 0 / 40 | 0 / 20 | 0 / 0 |
| *Ficedula zanthopygia* | 80 / 50 | 0 / 0 | 0 / 10 | 0 / 0 | 0 / 0 | 0 / 0 | 0 / 0 | 0 / 0 | 0 / 0 | 20 / 20 | 0 / 10 | 0 / 10 |
| *Fringilla montifringila* | 50 / 25 | 0 / 0 | 50 / 25 | 0 / 20 | 0 / 0 | 0 / 0 | 0 / 0 | 0 / 20 | 0 / 0 | 0 / 0 | 0 / 10 | 0 / 0 |
| *Geokichla sibirica* | 100 / 50 | 0 / 0 | 0 / 0 | 0 / 0 | 0 / 0 | 0 / 0 | 0 / 0 | 0 / 0 | 0 / 0 | 0 / 0 | 0 / 50 | 0 / 0 |
| *Halcyon coromanda* | 80 / 80 | 0 / 0 | 0 / 0 | 0 / 0 | 0 / 0 | 0 / 0 | 0 / 0 | 0 / 0 | 0 / 0 | 20 / 20 | 0 / 0 | 0 / 0 |
| *Hierococcyx hyperythrus* | 60 / 60 | 0 / 0 | 0 / 0 | 0 / 0 | 0 / 0 | 0 / 0 | 0 / 0 | 0 / 0 | 0 / 0 | 40 / 40 | 0 / 0 | 0 / 0 |
| *Hierococcyx sparverioides* | 100 / 50 | 0 / 0 | 0 / 10 | 0 / 0 | 0 / 0 | 0 / 0 | 0 / 0 | 0 / 0 | 0 / 0 | 0 / 20 | 0 / 20 | 0 / 0 |
| *Hirundapus caudacutus* | 40 / 60 | 0 / 0 | 30 / 0 | 10 / 40 | 10 / 0 | 0 / 0 | 0 / 0 | 0 / 0 | 10 / 0 | 0 / 0 | 0 / 0 | 0 / 0 |
| *Lanius tigrinus* | 30 / 40 | 0 / 0 | 30 / 0 | 0 / 0 | 0 / 0 | 0 / 0 | 0 / 0 | 30 / 30 | 0 / 0 | 0 / 0 | 10 / 30 | 0 / 0 |
| *Larvivora akahige* | 60 / 60 | 0 / 0 | 0 / 0 | 0 / 0 | 0 / 0 | 0 / 0 | 0 / 0 | 0 / 0 | 0 / 0 | 20 / 20 | 10 / 10 | 10 / 10 |
| *Larvivora cyane* | 50 / 40 | 0 / 0 | 50 / 30 | 0 / 0 | 0 / 0 | 0 / 0 | 0 / 0 | 0 / 0 | 0 / 0 | 0 / 30 | 0 / 0 | 0 / 0 |
| *Muscicapa dauurica* | 50 / 25 | 0 / 0 | 0 / 10 | 0 / 0 | 0 / 10 | 0 / 0 | 0 / 0 | 0 / 10 | 0 / 0 | 50 / 25 | 0 / 10 | 0 / 10 |
| *Muscicapa ferruginea* | 100 / 100 | 0 / 0 | 0 / 0 | 0 / 0 | 0 / 0 | 0 / 0 | 0 / 0 | 0 / 0 | 0 / 0 | 0 / 0 | 0 / 0 | 0 / 0 |
| *Muscicapa sibirica* | 100 / 100 | 0 / 0 | 0 / 0 | 0 / 0 | 0 / 0 | 0 / 0 | 0 / 0 | 0 / 0 | 0 / 0 | 0 / 0 | 0 / 0 | 0 / 0 |
| *Oriolus chinensis* | 30 / 30 | 0 / 0 | 10 / 10 | 0 / 0 | 0 / 0 | 0 / 0 | 0 / 0 | 15 / 15 | 0 / 0 | 15 / 15 | 15 / 15 | 15 / 15 |
| *Passer cinnamomeus* | 25 / 40 | 0 / 0 | 0 / 0 | 0 / 0 | 0 / 0 | 0 / 0 | 0 / 0 | 25 / 60 | 0 / 0 | 0 / 0 | 25 / 0 | 25 / 0 |
| *Pericrocotus divaricatus* | 60 / 50 | 0 / 0 | 0 / 0 | 0 / 0 | 10 / 10 | 0 / 0 | 0 / 0 | 10 / 10 | 0 / 0 | 10 / 10 | 0 / 0 | 10 / 20 |
| *Pernis ptilorhynchus* | 60 / 60 | 0 / 0 | 0 / 0 | 0 / 0 | 0 / 0 | 0 / 0 | 0 / 0 | 10 / 10 | 0 / 0 | 10 / 10 | 10 / 10 | 10 / 10 |
| *Phylloscopus borealis* | 50 / 20 | 0 / 0 | 50 / 20 | 0 / 20 | 0 / 0 | 0 / 0 | 0 / 0 | 0 / 20 | 0 / 0 | 0 / 0 | 0 / 20 | 0 / 0 |
| *Phylloscopus borealoides* | 100 / 40 | 0 / 0 | 0 / 0 | 0 / 0 | 0 / 0 | 0 / 0 | 0 / 0 | 0 / 0 | 0 / 0 | 0 / 0 | 0 / 30 | 0 / 30 |
| *Phylloscopus coronatus* | 100 / 50 | 0 / 0 | 0 / 50 | 0 / 0 | 0 / 0 | 0 / 0 | 0 / 0 | 0 / 0 | 0 / 0 | 0 / 0 | 0 / 0 | 0 / 0 |
| *Phylloscopus tenellipes* | 100 / 50 | 0 / 0 | 0 / 50 | 0 / 0 | 0 / 0 | 0 / 0 | 0 / 0 | 0 / 0 | 0 / 0 | 0 / 0 | 0 / 0 | 0 / 0 |
| *Phylloscopus xanthodryas* | 80 / 70 | 0 / 0 | 20 / 0 | 0 / 0 | 0 / 0 | 0 / 0 | 0 / 0 | 0 / 10 | 0 / 0 | 0 / 10 | 0 / 10 | 0 / 0 |
| *Pitta nympha* | 80 / 100 | 0 / 0 | 0 / 0 | 0 / 0 | 0 / 0 | 0 / 0 | 0 / 0 | 0 / 0 | 0 / 0 | 20 / 0 | 0 / 0 | 0 / 0 |
| *Tarsiger cyanurus* | 100 / 50 | 0 / 0 | 0 / 50 | 0 / 0 | 0 / 0 | 0 / 0 | 0 / 0 | 0 / 0 | 0 / 0 | 0 / 0 | 0 / 0 | 0 / 0 |
| *Terpsiphone atrocaudata* | 80 / 70 | 0 / 0 | 0 / 0 | 0 / 0 | 0 / 0 | 0 / 0 | 0 / 0 | 0 / 0 | 0 / 0 | 20 / 10 | 0 / 10 | 0 / 10 |
| *Turdus cardis* | 70 / 40 | 0 / 0 | 10 / 0 | 0 / 0 | 0 / 0 | 0 / 0 | 0 / 0 | 0 / 40 | 0 / 0 | 0 / 0 | 20 / 0 | 0 / 20 |
| *Turdus chrysolaus* | 50 / 20 | 0 / 0 | 50 / 20 | 0 / 0 | 0 / 0 | 0 / 0 | 0 / 0 | 0 / 20 | 0 / 0 | 0 / 0 | 0 / 20 | 0 / 20 |
| *Turdus hortulorum* | 50 / 50 | 0 / 0 | 50 / 50 | 0 / 0 | 0 / 0 | 0 / 0 | 0 / 0 | 0 / 0 | 0 / 0 | 0 / 0 | 0 / 0 | 0 / 0 |
| *Turdus obscurus* | 100 / 30 | 0 / 0 | 0 / 30 | 0 / 0 | 0 / 0 | 0 / 0 | 0 / 0 | 0 / 40 | 0 / 0 | 0 / 0 | 0 / 0 | 0 / 0 |
| *Turdus pallidus* | 40 / 25 | 0 / 0 | 30 / 0 | 0 / 0 | 0 / 0 | 0 / 0 | 0 / 0 | 0 / 25 | 0 / 0 | 0 / 0 | 0 / 25 | 30 / 25 |
| *Urosphena squameiceps* | 80 / 40 | 0 / 0 | 20 / 30 | 0 / 0 | 0 / 30 | 0 / 0 | 0 / 0 | 0 / 0 | 0 / 0 | 0 / 0 | 0 / 0 | 0 / 0 |

**Figure S1.** Annual rate of forest cover change of the three trend categories in (a) breeding and (b) non-breeding ranges breaking down to the five regions.

| a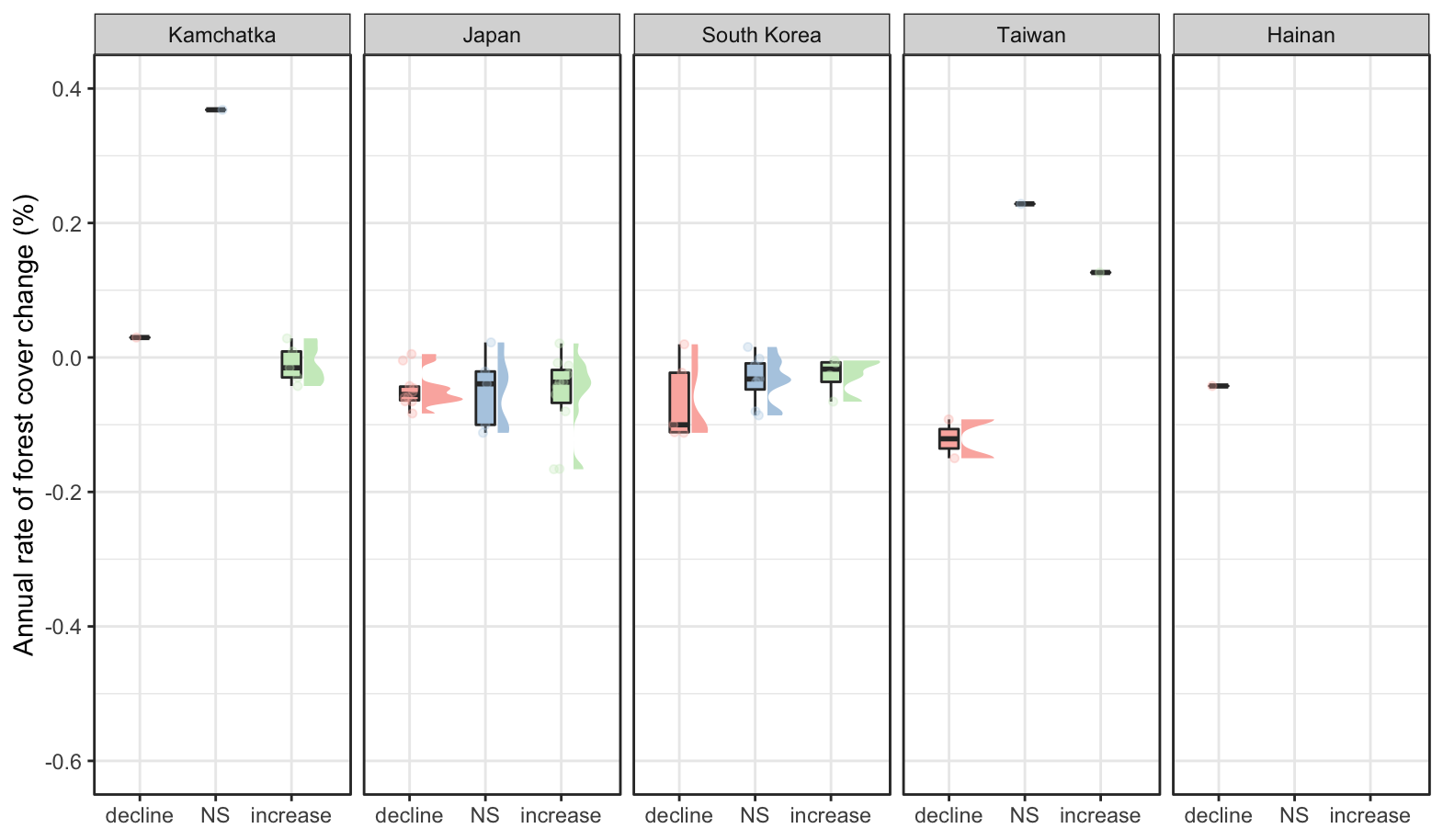 |
| --- |
| b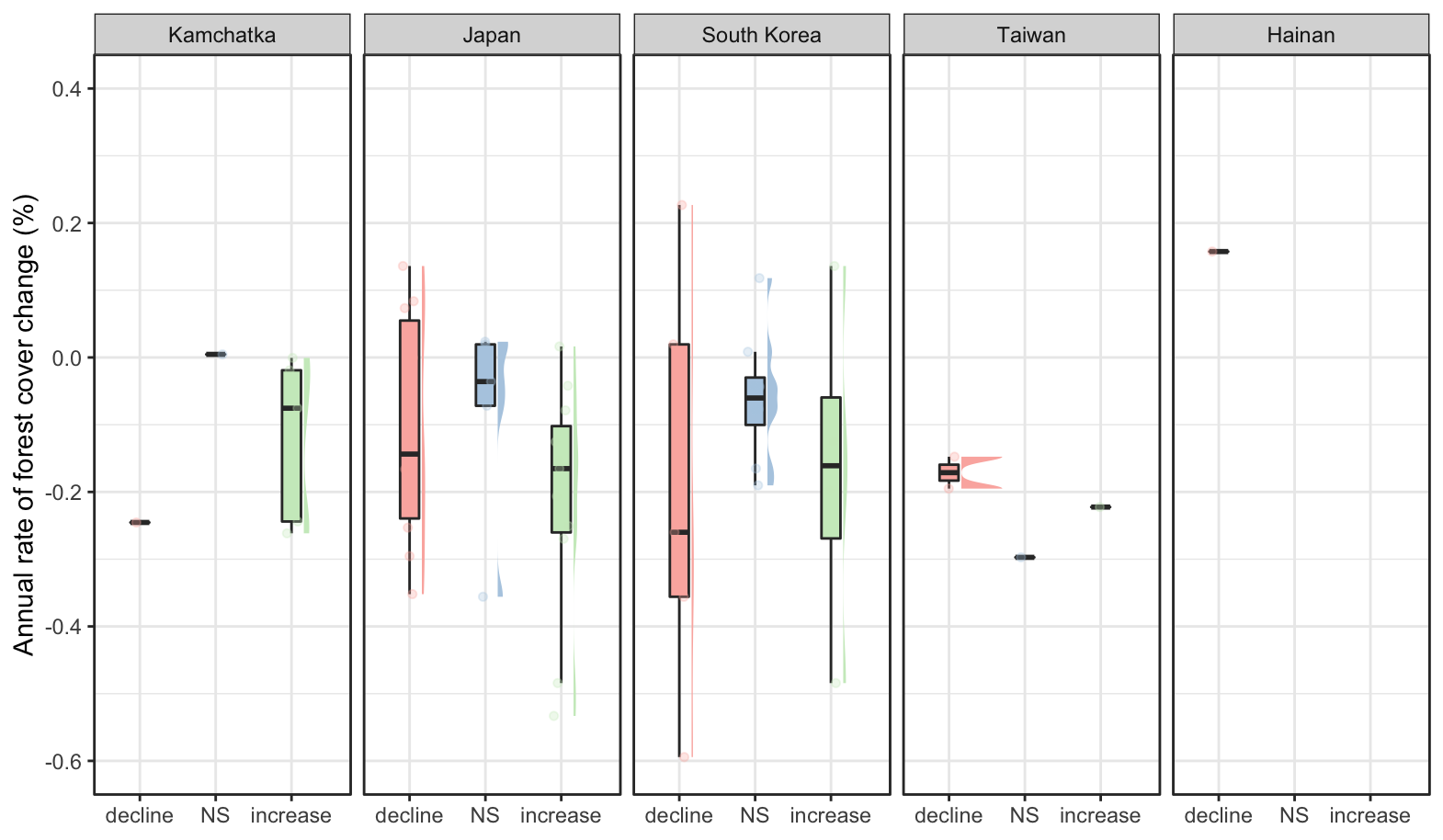 |

**Figure S2.** Scores of species’ forest preference (0-100) of the three trend categories in (a) breeding and (b) non-breeding seasons breaking down to the five regions.

| a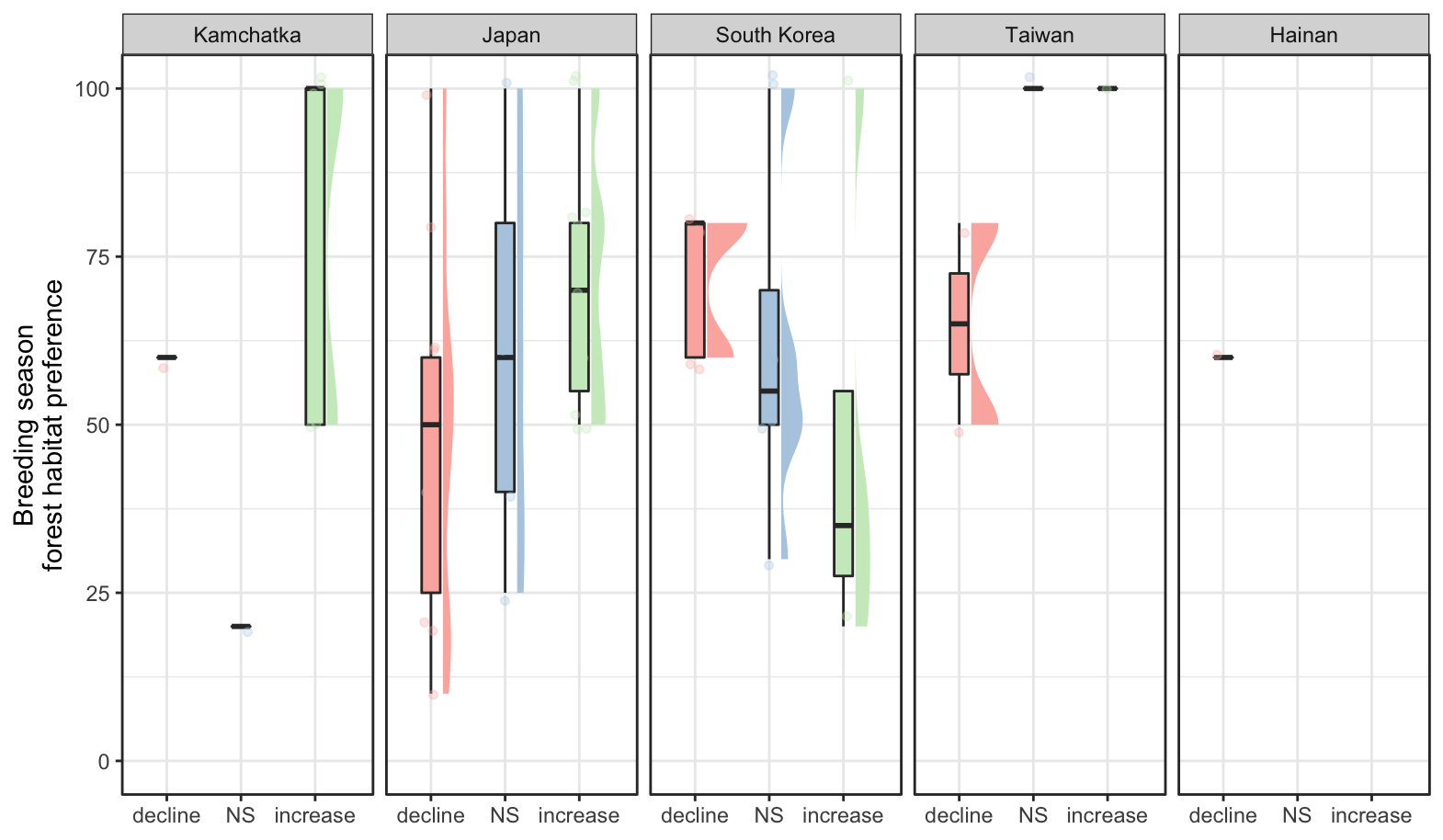 |
| --- |
| b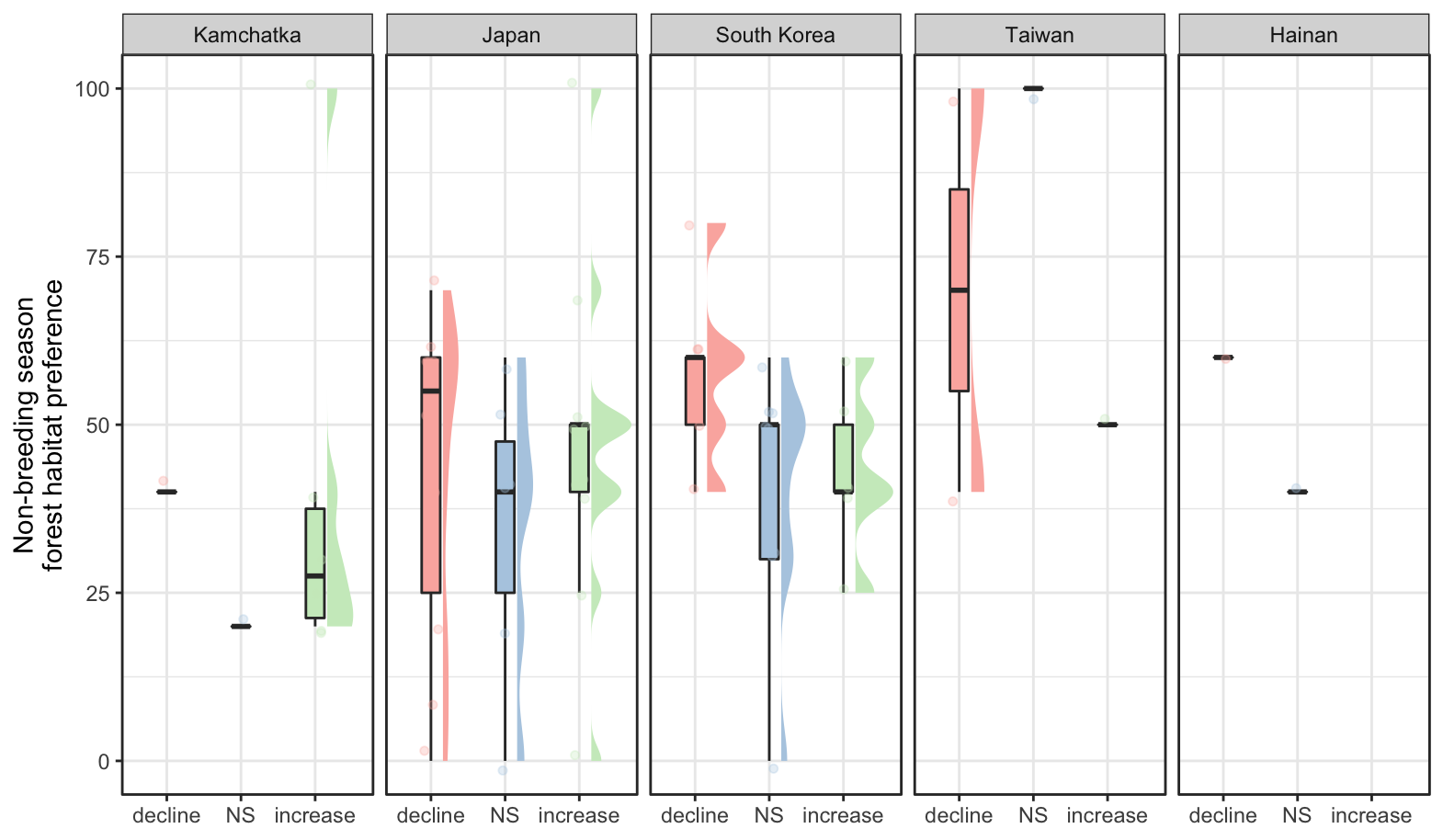 |

**Figure S3.** Estimated effects with 95% confidence intervals for predicting variables on the direction of regional population trend of migratory forest breeding birds in the EAF.

**
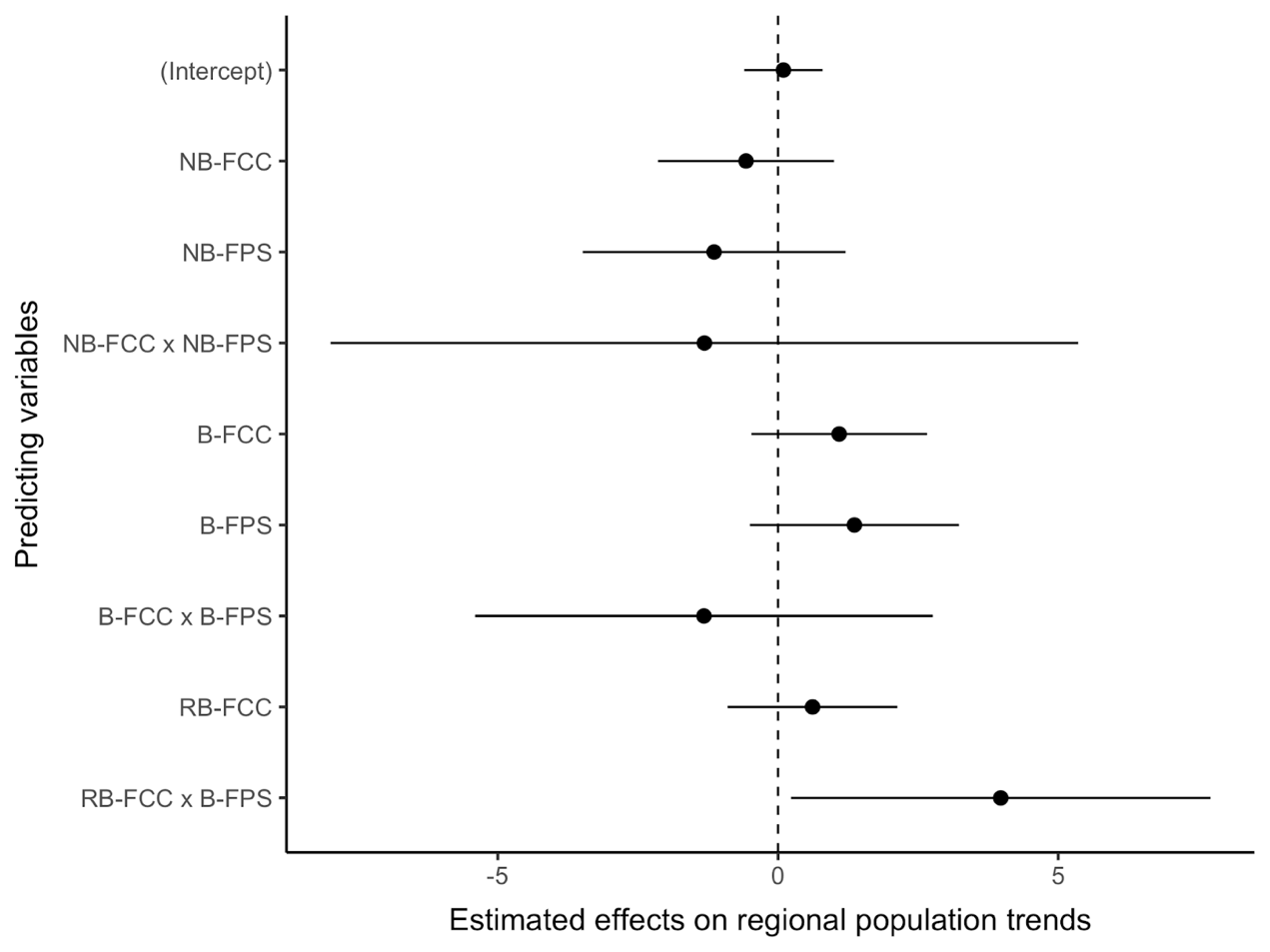
**
